# Psychometric properties of the abbreviated version of the Scale to Assess Unawareness in Mental Disorder in schizophrenia

**DOI:** 10.1186/1471-244X-13-229

**Published:** 2013-09-22

**Authors:** Pierre Michel, Karine Baumstarck, Pascal Auquier, Xavier Amador, Rémy Dumas, Jessica Fernandez, Christophe Lancon, Laurent Boyer

**Affiliations:** 1 Aix-Marseille University, EA 3279 – Public Health, Chronic Diseases and Quality of Life - Research Unit, 13284 Marseille, France; 2 Columbia University Teachers College and LEAP Institute, New York, USA; 3 Department of Psychiatry, Sainte-Marguerite University Hospital, 13009 Marseille, France

**Keywords:** Schizophrenia, Psychometric properties, Validity, Reliability, Insight, Scale to Assess Unawareness in Mental Disorder

## Abstract

**Background:**

The Scale to Assess Unawareness in Mental Disorder (SUMD) is widely used in clinical trials and epidemiological studies but more rarely in clinical practice because of its length (74 items). In clinical practice, it is necessary to provide shorter instruments. The aim of this study was to investigate the validity and reliability of the abbreviated version of the SUMD.

**Methods:**

*Design:* We used data from four cross-sectional studies conducted in several psychiatric hospitals in France. *Inclusion criteria:* a diagnosis of schizophrenia based on DSM-IV criteria. *Data collection:* socio-demographic and clinical data (including duration of illness, Positive and Negative Syndrome Scale, and the Calgary Depression Scale); quality of life; SUMD. *Statistical analysis:* confirmatory factor analyses, item-dimension correlations, Cronbach’s alpha coefficients, Rasch statistics, relationships between the SUMD and other parameters. We tested two different scoring models and considered the response ‘not applicable’ as ‘0’ or as missing data.

**Results:**

Five hundred and thirty-one patients participated in this study. The 3-factor structure of the SUMD (awareness of the disease, consequences and need for treatment; awareness of positive symptoms; and awareness of negative symptoms) was confirmed using LISREL confirmatory factor analysis for the two models. Internal item consistency and reliability were satisfactory for all dimensions. External validity testing revealed that dimension scores correlated significantly with all PANSS scores, especially with the G12 item (lack of judgement and awareness). Significant associations with age, disease duration, education level, and living arrangements showed good discriminant validity.

**Conclusion:**

The abbreviated version of the SUMD appears to be a valid and reliable instrument for measuring insight in patients with schizophrenia and may be used by clinicians to accurately assess insight in clinical settings.

## Background

Lack of insight is a prevalent feature that affects approximately 30 to 50% of patients with schizophrenia [1-4]. Impaired insight has been suggested as a predictive value for poor treatment responses and outcomes in patients with schizophrenia [1,5-7], especially by affecting a patient’s quality of life [8], adherence to treatment [9-11] and increasing the risk of relapse and re-hospitalisation [12,13]. On the other hand, several studies reported that high levels of insight can impair functioning, hope and quality of life. It has been suggested that these associations occur via self-stigma [14-16]. Because insight in schizophrenia is one important issue of pharmacological and psychological treatments, its assessment should be considered in the treatment and in the follow-up visits of patients. In recent years, researchers have reached a consensus on the definition of insight, which is now considered a continuous and multidimensional construct that includes the following aspects: (1) awareness of having a mental illness, (2) an understanding of the need for treatment, (3) awareness of the social consequences of mental disorders, (4) awareness of symptoms, and (5) attribution of symptoms to a mental disorder [1]. Among the various available tools, the Scale to Assess Unawareness in Mental Disorder (SUMD) is one of the most widely used instruments with satisfactory psychometric properties to measure insight, while considering the continuous and multidimensional approaches [17]. The SUMD is mainly used in clinical trials and epidemiological studies but more rarely in clinical practice because of its relative length (74 items) [5]. In clinical practice, it is necessary to provide shorter instruments, as is already the case for other measurements such as quality of life in schizophrenia [18]. Interestingly, an abbreviated version of the SUMD (9 items) has been used by the same authors in a large clinical study because of ‘interview time constraints’ [5]. This abbreviated version (see Additional file 1: Appendix) has not been validated using modern psychometric methods, commonly used for item reduction, in addition to those of the classical test theory (i.e., traditional psychometric methods such as principal component analysis and Cronbach’s alpha).

The purpose of this study was to investigate the validity and reliability of the abbreviated version of the SUMD. As a result of this investigation, clinicians will be able to accurately and consistently assess insight in their clinical practice.

## Methods

### Study population

We established a database for four studies carried out by members of the Schizophrenia Quality of Life Group, in which the SUMD was used to assess patient insight. The database included a total of 531 inpatients and outpatients recruited from one psychiatric hospital in Marseille, France. The inclusion criteria included the following factors: a diagnosis of schizophrenia according to the DSM-IV criteria [19], age over 18 years, informed consent to participate, and French as their native language. The exclusion criteria included the following factors: a diagnosis other than schizophrenia on Axis I of the DSM-IV, a decompensated organic disease and mental retardation. These studies were approved by the local and national ethics committees (Comité de Protection des Personnes Sud-Méditerranée I, France: trial number CM1-0512; Commission Nationale de l’Informatique et des Libertés: CNIL number: 00–1143) and were conducted in accordance with the Declaration of Helsinki and French Good Clinical Practices [20].

### Data collection

The data collected in the four different studies included socio-demographic information, clinical characteristics, and self-reported questionnaires. The data collected in each study are presented in Table 1. More specifically, the data collected included the following information:

1. Socio-demographic information: gender, age, living arrangement (alone or living with a partner/parents), and education level (primary/high school versus university level).

2. Clinical characteristics: duration of illness, psychotic symptoms based on the Positive and Negative Syndrome Scale (PANSS), which comprises three different subscales (positive, negative and general psychopathology) [21,22]; higher scores indicate more severe symptomatology. Another clinical characteristic is depression based on the Calgary Depression Scale for Schizophrenia (CDSS), which is a nine-item scale specifically designed for patients with schizophrenia that evaluates depression independently of extra-pyramidal and negative symptoms [23,24].

3. Quality of life was assessed using the S-QoL18 questionnaire [18]. The S-QoL is a specific, self-administered and multidimensional QoL questionnaire designed for people with schizophrenia and comprising 18 items [18,25]. Index score range from 0, indicating the lowest QoL, to 100, the highest QoL.

4. Insight was assessed by using the abbreviated version of the SUMD [5], which is a standardised expert-rating scale based on a patient interview and comprises 9 items (current awareness of the following states): 1. a mental disorder, 2. consequences of a mental disorder, 3. effects of drugs, 4. hallucinatory experiences, 5. delusional ideas, 6. disorganised thoughts, 7. blunted affect, 8. anhedonia, and 9. lack of sociability. Each item was encoded in the same way with respect to the following modalities: not applicable (response of ‘0’ or missing data), aware (response of ‘1’), slightly aware/unaware (response of ‘2’), and seriously unaware (response of ‘3’).

**Table 1:** Data collection and contents of the four databases

	**Study 1 (N=142)**	**Study 2 (N=113)**	**Study 3 (N=123)**	**Study 4 (N=153)**
Gender	x	x	x	x
Age	x	x	x	x
Disease duration	x	x	x	
Education level	x	x	x	x
Living arrangement	x	x	x	x
Symptomatology (PANSS)	x	x	x	x
Depression (CDSS)	x	x	x	
Quality of life (S-QoL18)	x	x	x	x
Insight (SUMD)	x	x	x	x

*PANSS* Positive and Negative Syndrome Scale, *CDSS* Calgary Depression Scale for Schizophrenia, *S-QoL 18* Schizophrenia Quality of Life, *SUMD* Scale to Assess Unawareness in Mental Disorder.

### Statistical analysis

#### Two scoring models

We tested two different models of scoring. As proposed by the authors of the SUMD [5], the first model of scoring (model 1) considered the response ‘not applicable’ to be ‘0’, thereby classifying an individual as less severe than an individual with a response of ‘1’ (aware). The second model of scoring (model 2) considered the response ‘not applicable’ as missing data, thereby classifying an individual as unconcerned. For model 2, data imputation was performed (due to the increased rate of missing data, which did not permit validation of the scale) considering data as missing not at random (MNAR) and using a method based on the Item Response Theory (IRT) models such as the one parameter logistic model [26]. This method determines the parameter estimate from the observed responses (completed items in the SUMD). The distribution of the responses for each cell in the data table was determined to be *π*_*i,j*_(*θ*_*v*_), or the estimated probability that the subject *S*_*v*_ with his true degree of insight *θ*_*v*_ gives the response *j* on item *I*_*i*_. The values used for the imputation of missing data were then drawn randomly from the estimated distribution of each cell, with the probabilities *π*_*i,*0_(*θ*_*v*_), … , *π*_*i,c*_(*θ*_*v*_), where *j* = 0, … , *c* are the *c* + 1 response options. Each missing value was imputed in this way. This procedure was then repeated five times to obtain a data table combining the five imputed data tables [27]. The item scores in these data tables were averaged.

The dimension scores were calculated using the mean scores of all items, and an index score was obtained using the mean values of the dimension scores. All of the scores were linearised on a scale of 0–100, with 100 representing the highest level of unawareness and 0 representing the lowest level of unawareness.

### Validation

The validation process was performed for the previously mentioned 2 models of scoring. The validation process included construct validity, reliability, and some aspects of external validity. The structure of the SUMD was explored using confirmatory factor analysis (LISREL model), previous studies having described a 3-factor structure of the SUMD (awareness of the disease, consequences and need for treatment; awareness of positive symptoms; and awareness of negative symptoms) [8,28]. The following indicators were required: the Root Mean Square Error of Approximation (RMSEA) is acceptable if <0.08 and satisfactory if <0.05, the Comparative Fit Index (CFI) and the General Fit index (GFI) are higher than 0.9, and the Standardised Root Mean Square Residual (SRMR) is closer to 0.

The unidimensionality of each dimension was assessed using a Rasch analysis. The goodness-of-fit statistics [inlier-sensitive fit (INFIT), ranging between 0.7 and 1.3] ensured that all items of the scale measured the same concept.

Internal structural validity was assessed using item-dimension correlations. An item’s internal consistency was assessed by correlating each item with its scale (corrected for overlap) using Pearson’s coefficient (a correlation of 0.4 was recommended for supporting item-internal consistency [29]; an item’s discriminant validity was assessed by determining the extent to which items correlate more highly with the dimensions they were supposed to represent [30]. For each dimension scale, reliability was assessed by using Cronbach’s alpha coefficient (a coefficient of at least 0.7 was expected for each scale [29]). Floor and ceiling effects were reported when assessing the homogeneous repartition of the response distribution. Proportions of missing values were provided (an acceptable rate was less than 15%). Inter-dimensional correlations were examined using Pearson’s and polychoric coefficients. Differential item functioning (DIF) analyses were performed, which compared the item differences between two groups of individuals according to socio-demographic parameters (gender, age, education level, disease duration, and living arrangement) to check whether all items behave the same way [31]. The DIF means that an item performs and measures differently for one subgroup of a population than for the other.

The external validity was assessed by studying the relationship between dimension scores of the SUMD and the scores of the other instruments (PANSS, CDSS, and S-QoL18). The discriminant validity was determined by comparing the SUMD dimension mean scores across patient groups (gender, education level, and living arrangement) and by studying the correlations of the SUMD dimension scores with age and disease duration.

Data analyses were performed using the PASW 17.0.2 computer software, Winsteps, Stata and LISREL software.

## Results

### Sample characteristics

Of the 531 subjects, the mean age was 38.2 years (standard deviation= 11.7), 67.8% were male, 38.8% had an education level above the university level, 34.8% were living alone and the average disease duration was 14.4 years (standard deviation= 9.6). These characteristics are presented in Table 2.

**Table 2:** Socio-demographic and clinical characteristics of the study sample (N=531)

		**Overall sample**	**Study 1 (N=142)**	**Study 2 (N=113)**	**Study 3 (N=123)**	**Study 4 (N=153)**
n (%) or M ± SD						
Gender (Male)		360 (67.8)	101 (71.1)	79 (69.9)	84 (68.3)	96 (62.7)
Age (in years)		38.2 ± 11.7	36.2 ± 12.7	38.5 ± 10.8	40.8 ± 11.5	37.6 ± 11.3
Disease duration (in years)		14.4 ± 9.6	12.3 ± 10.1	13.9 ± 8.1	17.1 ± 9.6	
Education Level (University level)		206 (38.8)	67 (55.8)	51 (45.1)	27 (22.0)	61 (43.0)
Living arrangement (Alone)		185 (34.8)	44 (37.9)	46 (40.7)	50 (41.3)	45 (36.3)
PANSS	Total score	69.2 ± 20.5	70.3 ± 19.9	63.9 ± 18.0	63.1 ± 21.6	77.3 ± 19.2
	Positive scale score	14.9 ± 6.1	14.5 ± 5.5	14.6 ± 6.2	12.9 ± 5.9	17.1 ± 6.0
	Negative scale score	17.8 ± 6.6	19.5 ± 7.3	15.8 ± 5.6	15.6 ± 6.3	19.4 ± 6.0
	General psychopathology score	36.6 ± 10.9	36.3 ± 10.1	33.5 ± 9.8	34.6 ± 11.5	40.8 ± 10.6
	G12	2.8 ± 1.5	3.1 ± 1.5	2.7 ± 1.4	2.4 ± 1.4	2.6 ± 1.1
CDSS	Total	4.2 ± 4.1	4.0 ± 4.2	4.1 ± 4.5	4.5 ± 3.6	
S-QoL18	Index	58.0 ± 18.9	57.7 ± 17.9	59.4 ± 19.4	55.0 ± 17.0	58.8 ± 20.4

*M ± SD* Mean ± Standard-Deviation.

*PANSS* Positive and Negative Syndrome Scale, *CDSS* Calgary Depression Scale for Schizophrenia, *S-QoL 18* Schizophrenia Quality of Life.

G12 General Psychopathology Scale: Lack of judgement and awareness of the disease.

### Validity of model 1 scoring: ‘not applicable’ = ‘0’

#### Construct validity and reliability

**Figure 1:**
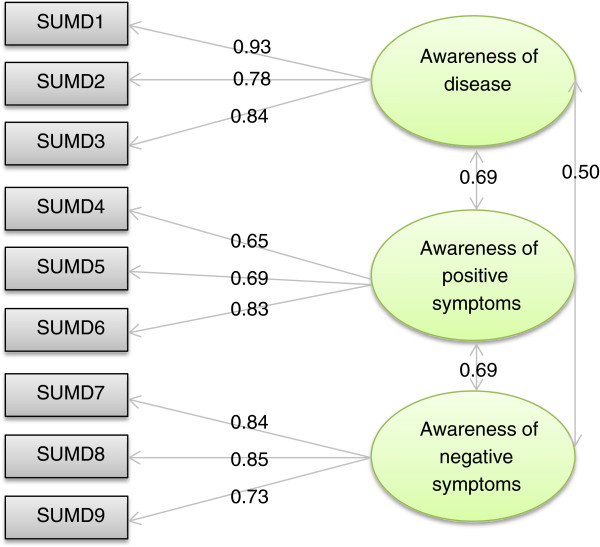
**LISREL confirmatory structural model (model 1, ‘not applicable’ = ‘0’).** SUMD1: Awareness of a mental disorder, SUMD2: Awareness of the consequences of a mental disorder, SUMD3: Awareness of the effects of drugs, SUMD4: Awareness of a hallucinatory experience, SUMD5: Awareness of delusional ideas, SUMD6: Awareness of disorganised thoughts, SUMD7: Awareness of blunted affect, SUMD8: Awareness of anhedonia, SUMD9: Awareness of lack of sociability.

A 3-factor structure was confirmed by confirmatory factor analysis. The dimensions were named according to their constitutive items: awareness of the disease, consequences and need for treatment (3 items), awareness of positive symptoms (3 items), and awareness of negative symptoms (3 items). This model showed a good fit, and all the indices from the confirmatory LISREL model were satisfactory (RMSEA=0.030, CFI=1.00, GFI=0.99, SRMR=0.018). The model is presented in Figure 1. The overall scalability was satisfactory; all of the items showed a good fit for the Rasch model in each dimension, and none of the items had a statistical INFIT outside the range of acceptability.

Item-internal consistency was satisfactory for all dimensions; each item achieved the 0.40 standard for item-internal consistency (ranging from 0.79 to 0.90). The correlation of each item with its contributive dimension was higher than with the other dimensions (item discriminant validity). Cronbach’s alpha coefficients ranged from 0.76 to 0.83, indicating satisfactory reliability. Floor effects ranged from 4.5 to 22.3%, and ceiling effects ranged from 11.4 to 17.1%. The percentage of missing data never exceeded 1%. According to the definition of the DIF, there should be no difference on the item behavior according to gender, age, living arrangement, disease duration, and education level. Inter-dimensional correlations were significant and ranged from 0.39 to 0.50 (data not shown).

All the dimension characteristics of the SUMD are provided in Table 3.

**Table 3:** Dimension characteristics of the SUMD

**Dimension/index (number of items)**	**M (SD)**	**Missing values %**	**Item-internal consistency (min-max)**	**Item discriminant validity (min-max)**	**Floor %**	**Ceiling %**	Alpha*	INFIT** (min-max)
***Model 1 scoring: ‘not applicable’ = ‘0’***								
Awareness of disease (3)	55.3 (22.1)	0.13	0.81–0.90	0.32–0.44	4.5	17.1	0.83	0.86–1.15
Awareness of positive symptoms (3)	47.2 (27.2)	0.70	0.79–0.85	0.37–0.48	22.3	15.3	0.76	0.88–1.09
Awareness of negative symptoms (3)	47.5 (25.4)	0.53	0.81–0.87	0.31–0.44	16.8	11.3	0.80	0.87–1.12
Index (9)	50.0 (20.0)	0.45	NA***	NA***	NA***	NA***	0.85	NA***
***Model 2 scoring: ‘not applicable’ = missing data***								
Awareness of disease (3)	56.20 (22.0)	1.6	0.82–0.93	0.43–0.70	48.2	17.1	0.87	0.73–1.39
Awareness of positive symptoms (3)	59.83 (19.6)	23.0	0.82–0.88	0.52–0.68	28.5	15.3	0.82	0.93–1.10
Awareness of negative symptoms (3)	57.45 (19.4)	15.5	0.85-0.90	0.43–0.67	35.2	11.4	0.85	0.81–1.14
Index (9)	57.83 (17.8)	13.4	NA***	NA***	NA***	NA***	0.85	NA***

*Cronbach’s Alpha, **Rasch’s statistics, ***NA Not Applicable.

M (SD) mean (standard deviation); a higher score represents a higher level of unawareness.

#### External validity

The SUMD scores showed low to medium significant correlations with the PANSS scores (correlation coefficients ranged from 0.25 to 0.55, all p<0.01). As expected, the ‘SUMD positive symptoms’ score correlated with the ‘PANSS positive’ score (R=0.44, p<0.01), the ‘SUMD negative symptoms’ score correlated with the ‘PANSS negative score (R=0.44, p<0.01), and the all the SUMD scores correlated with the specific item G12 of the PANSS, ‘lack of judgment and conscience of the disease’ (R from 0.33 to 0.67, p<0.01). SUMD scores were either poorly or not correlated with the depression scores and the QoL scores. All SUMD scores were poorly correlated with age (R from 0.13 to 0.17, p<0.01), and the two SUMD symptom scores were poorly correlated with disease duration (R from 0.11 to 0.12, p<0.01). No significant differences were reported regarding gender and living arrangement, except that the ‘awareness of positive symptoms’ score was significantly higher for the individuals who reported that they lived alone. Significantly higher levels of unawareness were found for individuals with lower education levels, except for the awareness of the disease, consequences and need for treatment score. All of the details are provided in Table 4.

**Table 4:** External validity of the SUMD dimension scores and index

		**Awareness of disease**	**Awareness of positive symptoms**	**Awareness of negative symptoms**	**Index**
***Model 1 scoring: ‘not applicable’ = ‘0’***					
PANSS	Total score	0.395**	0.482**	0.436**	0.550**
	Positive score	0.261**	0.437**	0.251**	0.402**
	Negative score	0.373**	0.348**	0.440**	0.483**
	General psychopathology score	0.373**	0.454**	0.415**	0.521**
	G12	0.677**	0.471**	0.333**	0.592**
CDSS total score		−0,019	0.098	0.139**	0.095
S-QoL18 Index		0.009	−0.091	−0.180*	−0.115*
Age		0,132**	0.149**	0.134**	0.173**
Disease duration		0,032	0.112*	0.116*	0.109
Gender	Male	55.79 (22.2)	46.47 (27.7)	46.80 (25.2)	49.68 (19.9)
	Female	54.13 (22.0)	48.70 (26.1)	48.86 (25.8)	50.56 (20.2)
	p-value	0.419	0.377	0.382	0.634
Education level	University level	53.45 (21.9)	43.07 (27.2)	42.98 (25.5)	46.50 (20.1)
	High school/primary school level	55.61 (21.9)	49.89 (26.7)	50.63 (24.9)	52.04 (19.7)
	p-value	0.278	**0.006**	**0.001**	**0.002**
Living arrangement	Alone	55.62 (21.3)	50.87 (27.7)	47.33 (25.8)	51.27 (20.4)
	Not alone	53.96 (22.1)	43.91 (26.7)	46.49 (25.8)	48.11 (19.7)
	p-value	0.420	**0.007**	0.731	0.094
***Model 2 scoring: ‘not applicable’ = missing data***					
PANSS	Total score	0.389**	0.465**	0.365**	0.465**
	Positive score	0.260**	0.355**	0.290**	0.344**
	Negative score	0.370**	0.428**	0.382**	0.450**
	General psychopathology score	0.364**	0.420**	0.296**	0.413**
	G12	0.676**	0.682**	0.501**	0.697**
CDSS total score		−0.007	0.045	−0.058	−0.007
S-QoL18 Index		0.001	0.001	0.010	0.005
Age		0.124**	0.112**	0.162**	0.152**
Disease duration		0.035	−0.002	0.074	0.039
Gender	Male	56.87 (22.0)	60.49 (19.6)	57.42 (19.0)	58.26 (17.4)
	Female	54.81 (22.1)	58.45 (19.6)	57.50 (20.5)	56.92 (18.5)
	p-value	0.315	0.264	0.962	0.419
Education level	University level	54.34 (22.3)	58.03 (19.8)	54.52 (18.7)	55.63 (17.8)
	High/Primary school level	56.66 (21.6)	60.56 (19.4)	59.25 (19.3)	58.82 (17.6)
	p-value	0.244	0.156	**0.007**	**0.047**
Living arrangement	Alone	56.94 (21.1)	62.17 (19.8)	59.06 (18.8)	59.39 (17.4)
	Not alone	54.73 (22.3)	57.82 (19.4)	56.52 (19.6)	56.36 (17.8)
	p-value	0.283	**0.018**	0.164	0.069

G12 General Psychopathology Scale: Lack of judgement and awareness of the disease.

*PANSS* Positive and Negative Syndrome Scale, *CDSS* Calgary Depression Scale for Schizophrenia, *S-QoL 18* Schizophrenia Quality of Life.

Bold values p<0,05, *p<0.05, **p<0.01.

### Validity of model 2 scoring: ‘not applicable’ = missing data

#### Construct validity and reliability

**Figure 2:**
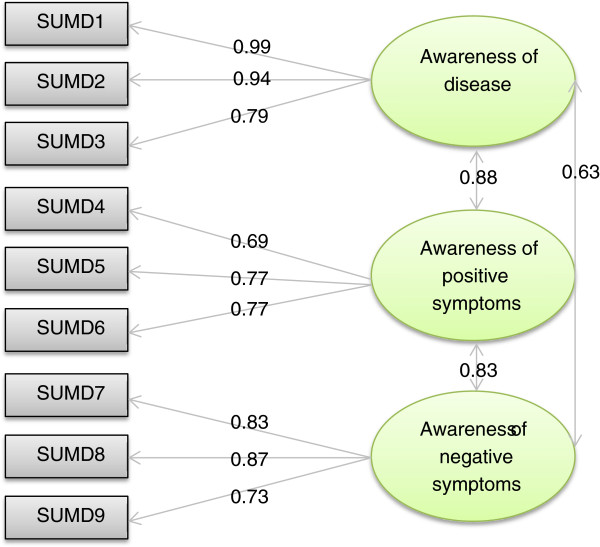
**LISREL confirmatory structural model (model 2, data imputation).** SUMD1: Awareness of a mental disorder, SUMD2: Awareness of the consequences of a mental disorder, SUMD3: Awareness of the effects of drugs, SUMD4: Awareness of a hallucinatory experience, SUMD5: Awareness of delusional ideas, SUMD6: Awareness of disorganised thoughts, SUMD7: Awareness of blunted affect, SUMD8: Awareness of anhedonia, SUMD9: Awareness of lack of sociability.

The 3-factor structure was retrieved. The LISREL indicators were satisfactory (RMSEA=0.035, CFI=1.00, GFI=0.99, SRMR=0.015). The LISREL model is presented in Figure 2.

The overall scalability was satisfactory; all items showed a good fit for the Rasch model in each dimension, and none of the items had a statistical INFIT outside the range of acceptability. We also noted the absence of a uniform differential item functioning (DIF) by gender, age, living arrangement, disease duration, or education level.

Item-internal consistency was satisfactory for all dimensions; each item achieved the 0.40 standard for item-internal consistency, and the correlation with its associated contributive dimension was higher than the correlation with the other dimensions. The reliability was satisfactory (Cronbach’s alpha coefficients > 0.70). Floor effects ranged from 28.5% to 48.2%, and ceiling effects ranged from 11.4% to 17.1%. The missing data rate ranged between 1.6% and 23.0% for the dimension scores and 13.4% for the index. All of the details are provided in Table 3.

#### External validity

The results are similar to those found in model 1. The SUMD scores showed low to high significant correlations with the PANSS scores (correlation coefficients ranged from 0.26 to 0.68, all p<0.01). All of the SUMD scores were highly correlated with the specific item G12 of the PANSS (R from 0.50 to 0.68, p<0.01). The SUMD scores were not correlated with the depression and QoL scores. All SUMD scores were correlated with age, while no SUMD scores correlated with the disease duration. Regarding model 1, no significant differences were reported according to gender and living arrangement (except that the ‘awareness of positive symptoms’ score was significantly higher for individuals who reported that they lived alone). Significantly higher levels of unawareness were found for individuals with lower education levels for the awareness of negative symptoms index. All of the details are provided in Table 4.

## Discussion

In this study, we demonstrated the validity and reliability of the abbreviated version of the SUMD in a series of analyses. The internal structure, which was supported by a high internal consistency, confirmed that patient insight is a multidimensional concept [1]. As described in previous studies [8,28], the 3 dimensions of the SUMD (i.e. awareness of the disease, consequences and need for treatment; awareness of positive symptoms; and awareness of negative symptoms) were confirmed by LISREL model. Moreover, the internal consistency reliability of the three dimensions was proven to be high. External validity, which was explored through the use of socio-demographic characteristics and established psychiatric and QoL measures, generally confirmed assumptions made by previous researchers. As in our findings, a lack of insight has been associated with prolonged illness duration [32] and an increased severity of positive and negative symptoms [5,33-36]. Older patients presented a lesser degree of insight regardless of disease duration (data not shown, no interaction between age and disease duration) suggesting a specific effect of age on insight. However, previous studies have also reported that older individuals have better insight [37,38]. In addition, we found a link between a lack of insight and lower education levels, as was also found in several previous studies [35,39-41]. This finding suggests that more-educated patients are more likely to be better equipped to make accurate self-assessments and to evaluate their illness [39]. Concerning the relationship between insight and QoL, existing studies have revealed contradictory results. As previously determined [42-45], we found higher QoL scores for patients who presented higher levels of insight. Other reports, however, have found either no relationship between QoL and insight [46-48] or an inverted relationship between these two factors [49-52] Finally, DIF analyses were satisfactory and revealed a noteworthy property that has rarely been studied in other questionnaires. We speculate that items of the SUMD have equivalent measurement properties according to the patients’ characteristics (i.e. the same probability to answer a same response to an item, for a same level of underlying insight). Therefore, the abbreviated version of the SUMD met the standards for psychometric properties, suggesting that this shortened scale may be both appropriate and useful for research and clinical practices.

The validation process of the SUMD poses serious problems for psychometric analyses because not all items are rated for every patient. As a consequence, missing values (MVs) are frequent and make it difficult to perform data analysis. Inappropriate handling of the MVs in the analysis may bias the results and can suggest misleading conclusions being drawn from a research study. It may also limit one’s ability to generalise the research findings [53]. We thus treated the MVs in two different ways to guarantee the robustness of our results. Model 1 considered the response ‘not applicable’ to be ‘0’ (less severe than an individual with a response of ‘1’ - aware). Although replacing ‘not applicable’ with 0 was proposed by the authors of the SUMD [5], this choice may be criticised because 0 does not correspond to the measure of insight but rather to the absence or presence of symptoms. We have thus chosen to remove zero values and replace them with MVs. MVs were particularly high for awareness of positive symptoms (23%), awareness of negative symptoms (16%), and the index (13%), which constituted problems in terms of construct validity and reliability testing of the scale [54]. Therefore, we considered data imputation methods using the Item Response Theory models to treat the present MVs before applying the classification methods. MVs were filled in with estimated ones based on the observed responses for the other items of the SUMD. The validation process, including construct validity, reliability, and some aspects of external validity, was performed for the 2 models, and the indicators of these two models generally matched, thereby confirmed the robustness of our findings. These two models with 0 or MVs appear valid and may be used alternatively by clinicians and researchers. A short statement about the description of the model should also be included in each study to avoid confusion in the interpretation of scores.

Finally, one feature of the abbreviated version of the SUMD, in comparison to the longer version, is its narrower definition of insight, which does not assess the attribution phenomenon. Indeed, the long version is of particular interest because of its multidimensional approach to defining insight and its detailed assessment of patients’ awareness of and attributions for a wide range of signs and symptoms [17]. The long version of the SUMD is one of the longest instruments (74 items) among various insight measures, including the insight and treatment attitude questionnaire (ITAQ, 11 items) [2], the schedule for the assessment of insight (SAI, 3 items) [1], the positive and negative syndrome scale (PANSS, 1 item) [21], the Soskis scale (6 items) [55], the self-report insight scale for psychosis (ISP, 3 items) [56], and the insight scale (IS, 32 items) [57,58]. According to several authors, a short form of a scale is frequently associated with better acceptability in clinical practices [59]. The abbreviated version of the SUMD (9 items) may appear to be more practical than the long version and could lead to the inclusion of insight assessments as a part of routine clinical practice to offer individualised care.

### Strengths and limitations

There are several strengths and limitations of this study.

The large size of our sample (N = 531) may better guarantee the robustness of the instrument validation results across the large spectrum of patients with schizophrenia. Although a large number of insight instruments have already been validated for patients with schizophrenia, their process of validation has often used small study samples. For example, the validation of the long version of the SUMD was initially conducted using 43 patients [17] and more recently using 100 patients [60]. The ITAQ was validated among 52 patients [2], the ISP among 30 patients [56], and the IS among 43 [57] and 64 patients [58]. However, even with the large overall sample size used in this study, one may question whether it is representative of the patient population because participants were recruited only in France.

Several psychometric properties were not tested. In particular, inter-judge reliability is an important property for expert-rating scales such as the SUMD. Previous studies have, however, shown a satisfactory inter-judge reliability for the long version of the SUMD [17,60]. Sensitivity to change is also of particular interest for patient follow-ups in clinical practices and should thus be explored in future studies.

Finally, an important aspect of our study was the choice of our method to treat MVs. Indeed, a variety of mechanisms and methods to handle missing data exists and could be used. First, three data mechanisms have been proposed [61]: missing at random (MAR), missing completely at random (MCAR), and missing not at random (MNAR). Our choice to consider a MNAR mechanism may be criticised by other researchers. However, the Little’s MCAR test obtained for our dataset indicated that the data was not missing at random (data not shown, p < 0.001) [62]. In addition, we felt that our choice was more appropriate because the missing data depend on the patient’s health status, and these data may thus be considered not random [63]. On the other hand, several methods to impute data are also available. The classical approach to imputing data is imputation by mean score, which is a technique known to be inefficient when the rate of missing data is too high, as shown in our study [64]. We therefore chose to use a IRT model-based multiple imputation technique, which yielded more robust and unbiased results [63].

## Conclusion

The abbreviated version of the SUMD is not intended to replace the long version of the SUMD, which proposes a more detailed assessment of patient insight. The abbreviated version of the SUMD appears to be a practical, valid and reliable instrument for measuring insight in patients with schizophrenia and may be used by clinicians to accurately and consistently assess insight in a clinical setting.

## Competing interests

The authors have declared that there are no competing interests regarding the subject of this study.

## Authors’ contributions

Conception and design: PM, KB, PA, XA, LB. Study’s coordination: RD, CL. Inclusion and clinical data collection: RD, CL. Analysis of data: PM, JF. Interpretation of data: PM, KB, PA, XA, CL, LB. Drafting and writing the manuscript: PM, KB, PA, LB. All authors read and approved the final manuscript.

## Pre-publication history

The pre-publication history for this paper can be accessed here:

http://www.biomedcentral.com/1471-244X/13/229/prepub

## Supplementary Material

Additional file 1 **Appendix.** The abbreviated version of the Scale to Assess Unawareness in Mental Disorder in schizophrenia. Click here for file
