# Supplementary material for: Psychometric properties of the abbreviated version of the Scale to Assess Unawareness in Mental Disorder in schizophrenia
Source: BMC Psychiatry. 2013 Sep 22;13:229. doi: 10.1186/1471-244X-13-229 (PMC3851247; doi:10.1186/1471-244X-13-229)
Supplement: Additional file 1 — Appendix. The abbreviated version of the Scale to Assess Unawareness in Mental Disorder in schizophrenia. [file 1471-244X-13-229-S1.docx]

Additional file 1: Appendix

**The abbreviated version of the Scale to Assess Unawareness in Mental Disorder in schizophrenia**

| **1. Awareness of mental disorder:** In the most general terms, does the subject believe that he or she has a mental disorder? |
| --- |
| **2. Awareness of the consequences of mental disorder:** What is the subject’s belief regarding the reason(s) he or she has been unemployed, evicted, hospitalized, etc.? |
| **3. Awareness of the effects of drugs:** Does the subject believe that medications have diminished the severity of his or her symptoms (if applicable) ? |
| **4. Awareness of hallucinatory experiences:** Does the subject believe that he or she experiences hallucinations as such? Rate his or her ability to interpret this experience as primarily hallucinatory. |
| **5. Awareness of delusional ideas:** Does the subject believe that he or she experiences delusions as such, that is, as internally produced erroneous beliefs ? Rate his or her awareness of the implausibility of the belief if applicable. |
| **6. Awareness of disorganized thoughts:** Does the subject believe that his or her communications are disorganized? |
| **7. Awareness of blunted affect:** >Rate the subject’s awareness of his or her affect as communicated by his or her expressions, voice, gestures, etc. Do not rate his or her evaluation of his or her mood. |
| **8. Awareness of anhedonia:** Is the subject aware that his or her behaviour reflects an apparent decrease in experiencing pleasure while participating in activities normally associated with such feelings? |
| **9. Awareness of lack of sociality:** Is the subject aware that he or she shows no interest in social relationships? |
